# EnsembleFam: towards more accurate protein family prediction in the twilight zone

**DOI:** 10.1186/s12859-022-04626-w

**Published:** 2022-03-14

**Authors:** Mohammad Neamul Kabir, Limsoon Wong

**Affiliations:** Department of Computer Science, National University of Singapore, 13 Computing Drive, 117417 Singapore, Singapore

**Keywords:** Protein function prediction, Twilight zone sequence, Sequence homology, Support vector machine, Ensemble classifier

## Abstract

**Background:**

Current protein family modeling methods like profile Hidden Markov Model (pHMM), *k*-mer based methods, and deep learning-based methods do not provide very accurate protein function prediction for proteins in the twilight zone, due to low sequence similarity to reference proteins with known functions.

**Results:**

We present a novel method EnsembleFam, aiming at better function prediction for proteins in the twilight zone. EnsembleFam extracts the core characteristics of a protein family using similarity and dissimilarity features calculated from sequence homology relations. EnsembleFam trains three separate Support Vector Machine (SVM) classifiers for each family using these features, and an ensemble prediction is made to classify novel proteins into these families. Extensive experiments are conducted using the Clusters of Orthologous Groups (COG) dataset and G Protein-Coupled Receptor (GPCR) dataset. EnsembleFam not only outperforms state-of-the-art methods on the overall dataset but also provides a much more accurate prediction for twilight zone proteins.

**Conclusions:**

EnsembleFam, a machine learning method to model protein families, can be used to better identify members with very low sequence homology. Using EnsembleFam protein functions can be predicted  using just sequence information with better accuracy than state-of-the-art methods.

## Background

As next-generation sequencing technologies are becoming more affordable and faster, millions of protein sequences are derived within a very short time [1]. Although biological and molecular experiments are the gold standard for annotating proteins with their functions, these experiments are low throughput and also resource-demanding [2]. Thus, experimentally verified functional annotation of proteins is far behind the number of sequenced proteins.

Many computational approaches have been developed to annotate proteins. These approaches try to infer the function of an unknown protein by comparing it with reference proteins having known functions. Two protein sequences can easily be compared using local sequence alignment, but the task becomes difficult when the sequences are distantly related. To solve this problem, protein sequences with similar biomolecular functions are put together in a family, so that their shared features can be computationally more easily identified and modeled. Most of the computational methods perform well for proteins that have moderate to high similarity with reference proteins of known function. However, these methods do not perform well for the so-called twilight-zone proteins [3, 4], which are remote homologs with low sequence similarity to reference proteins of known function. Even though this difficulty was identified more than two decades ago, recent approaches still cannot produce good enough results comparable to that of high-similarity proteins. For example, INGA [5], a protein function prediction tool, works only with sequences having $40\%$ sequence identity or more; HHSearch [6] and LOMETS [7] both experimented against twilight zone sequences (identity $<20\%$ for HHSerach and identity $<25\%$ for LOMETS) to analyze performance, but the performance reported by both methods is still a lot poorer compared to higher similarity region; QAUST [8] tried to address this problem using multiple information sources; etc. This implies that predicting function for twilight zone protein is still a difficult computational problem.

Current protein family modeling methods can be roughly divided into three categories: sequence homology-based methods, alignment-free methods, and machine learning-based methods. These are briefly described below.

### Sequence homology-based methods

Protein sequence homology is the sequence similarity due to ancestry between proteins. While protein sequences may change in the course of evolution, the homologous segments (i.e. the segments conserved by evolution) are responsible for bio-molecular function with some exceptions (i.e. in some cases homologous segments may be responsible for different function) [9–11]. Many methods have been developed for detecting sequence similarity. The Smith–Waterman algorithm [12] based on dynamic programming is one of the earliest and more fundamental methods. When used with an affine gap penalty, this algorithm has cubic time complexity with respect to sequence length and thus is inefficient for comparing a query protein sequence to a large database of reference sequences. So methods such as BLAST [13] use heuristics to selectively compare a test protein sequence to only a subset of reference sequences in the database, where the subset consists of reference sequences having a sufficient number of exact short substring matches to the test sequence. Although pairwise sequence alignment provides valuable information, it is still a difficult task to accurately predict protein functions from these alignments. Hence, approaches that model an entire protein family based on multiple sequence alignment of the family were introduced. The pHMM (profile Hidden Markov Model) [14] is a very successful example of these, and the popular protein family database Pfam [15] uses pHMM to model the families. Other methods based on sequence alignment information include GOtcha [16] which uses term-specific probabilities to predict proteins from sequence alignment, GOblet [17] which provides different databases to choose and align sequences with a user-specified threshold to annotate unknown sequence, and OntoBlast [18] which provides a weighted list of sequences of a similar function using BLAST search.

### Alignment-free methods

Researchers have also developed alignment-free methods to annotate protein sequences. One of the approaches is to use word frequency of amino acids in the sequences as features to model the families [19]. Another strategy that uses oligomer distances as features along with remote homology detection, shows better performance than some alignment-based methods [20]. There are also many methods that use additional information, such as protein-protein interaction data, to annotate proteins [21–23].

### Machine learning-based method

Predicting protein function only from protein sequences, without using any other type of information is challenging. Many machine learning-based methods have been developed in this regard. One such example is SVM-Prot [24], where structural and residue properties such as amino acid decomposition, hydrophobicity, polarity, etc. of a protein constitute the feature vector to train an SVM classifier. A more interesting example is SVM-Fisher [25] which couples an iterative profile HMM training scheme to an SVM, where the vector of profile HMM gradients of a protein is used as the feature vector for training the SVM. A refinement of this, with much better performance, is SVM-pairwise [26], where given a reference set of protein sequences, the vector of pairwise sequence similarity scores of a protein to each of the reference proteins is instead used as the feature vector for training SVM classifiers for each protein family. Besides SVM, *k*-nearest neighbour (e.g. MS-kNN [27]), gradient tree boosting (e.g. PredSAV [28]) and other machine learning methods have also been used. Another popular method, CATH FunFam [29], uses structure and sequence information to predict function domains. Along with these, the Continuous Assessment of Functional Annotation (CAFA) [30] competition also introduces many different function prediction methods to predict Gene Ontology [31] terms. Most of the top methods in the competition are based on machine-learning and show great performance. A few examples of top methods from CAFA [30] are GOLabeler [32], PANNZER [33], INGA [5], FunFam [34], etc.

More recently, deep learning methods have been used for protein function prediction. An example is ProLanGO [35], which treats protein function prediction as a language translation problem, where a protein is mapped to a sequence of words in a “protein language” ProLan, and then translated to a “protein function language” GOLan using three layers of specially trained recurrent neural networks (RNNs). UDSMProt [36] is another recent method, which uses similar language modeling task with a pre-trained RNN model, and can be applied for enzyme class prediction, gene ontology prediction and fold detection from unlabeled protein sequence. An example which appears to have a much more impressive performance in protein family prediction is DeepFam [37], which uses a convolutional neural network (CNN) to extract high-level features from amino acid sequence.

### Limitations of the current approaches

Sequence homology-based methods suffered from two shortcomings. The first is that sequence alignment is too inefficient for comparing a query protein sequence to a large database of reference sequences. The second is that, when heuristics are used to select a small subset of the database, as in BLAST, there is a large reduction in sensitivity, as test sequences that are remote homologs to reference sequences in that database often do not have sufficient numbers of exact short matches to these reference sequences.

Alignment-free methods also have their shortcomings. Firstly, these methods typically require exact matches of *k*-mers, but remote homologs may not have many of these; this affects sensitivity. Secondly, alignment-free methods do not take into account the order of *k*-mers in the protein sequences; this loses biological information and affects specificity. Thirdly, finding the optimal value of *k* for *k*-mer based methods is another challenge. In general, alignment-free methods do not perform as well as alignment-based methods.

Although machine learning-based methods provide good results, they have weaknesses. For example, in SVM-Pairwise, the size of the feature vector is the number of reference protein sequences, which is a very large number. This puts severe demand on memory size during training and for storing the models of each protein family. It is also time-consuming to generate the feature vector of a query sequence, which makes the prediction slower. Moreover, totally novel query sequences and query sequences that are very different in length from the reference sequences require special treatment. For DeepFam, which is a multi-class classifier, the number of classes is fixed and each query sequence must be assigned to one of these classes. If a query sequence is from a new family, DeepFam [37] will wrongly force it to one of the trained classes. And in the case of ProLanGO [35], the protein sequences are modeled using a machine translation model which is popularly used for natural languages. But protein sequences differ from a natural language in many aspects. Moreover, in general, deep learning models have a large number of parameters to fit. Protein families with fewer training sequences cannot be modeled well using deep learning.

Other than these methods, some methods use information from different sources and combine them to make a final prediction for protein functions. For example, QAUST [8] uses structure similarity, protein-protein interaction, and sequence information to determine protein functions; INGA [5] uses protein interaction networks, sequence similarity and domain assignments to make prediction; etc. Methods like GOLabeler [32] works with comparatively lower similarity sequences (having sequence identity of $< 60\%$), which may include twilight zone proteins along with sequences having decent enough similarity. QAUST [8] focuses on twilight zone proteins, but all the required information (such as PPI data) for this method may not be readily available for many query proteins, which can lead to a poor prediction outcome. Thus, a computational method that can improve prediction for twilight zone proteins with minimum information provided, should be sought.

### Our approach

We introduce here EnsembleFam, a protein family modeling approach inspired by SVM-Pairwise. In SVM-Pairwise, for every protein sequence, its pairwise similarity scores to every reference protein sequence are used to form its feature vector; this makes the feature vector huge and time-consuming to produce. EnsembleFam differs from SVM-Pairwise in two important ways. Firstly, instead of calculating pairwise similarity with all reference sequences, the similarity scores are calculated per protein family. Thus the size of the feature vector used by EnsembleFam is orders of magnitude smaller than that of SVM-Pairwise and is much more efficient to produce. Secondly, EnsembleFam trains multiple SVM models for each family and makes a final prediction by ensembling individual predictions. As a result, EnsembleFam is much more sensitive on twilight zone proteins, while being highly competitive on easier proteins. Lastly, especially when compared to other machine learning methods, EnsembleFam has means for distinguishing members of new unknown protein families from members of the reference protein families.

## Results

### Dataset

To assess the performance of EnsembleFam we used two datasets namely Clusters of Orthologous Groups of proteins (COG) dataset and G Protein-Coupled Receptor (GPCR) dataset. These two datasets are widely used and have different characteristics. Although the Pfam database [15] is one of the most popular protein family databases, it was not adopted for this study due to its bias towards the protein Hidden Markov Model (pHMM).

#### Clusters of orthologous groups of proteins dataset

COG is one of the most extensively used functional databases. We used the latest version of the database which is made public in 2014 [38]. The protein family assignment in the COG database is done by pairwise sequence comparison in the whole-genome context. The functional annotation in this database should be reliable as the functional curation for clusters was done manually.

**Table 1:** Detailed description of the three subsets of the COG database based on threshold number of members in each family

Name	Min no. of members	No. of families	No. of proteins
COG-500-1074	500	1074	1,129,428
COG-250-1796	250	1796	1,389,595
COG-100-2892	100	2892	1,565,976

In the COG database different family consists of a different number of proteins which varies over a very high range. As we compare our method with DeepFam [37], we filtered the database in the same way so that it can be compared with DeepFam. For this, the sequences longer than 1000 amino acids were filtered away as DeepFam requires a fixed length for all proteins. EnsembleFam can work with a variable length of proteins without any restrictions. There are in total 4655 protein families with 1,674,176 proteins after removing the longer sequences. Furthermore, the database is divided into three different subsets based on the minimum number of sequences in one family. The three thresholds used for this filtration are 100, 250, and 500. Therefore, the three sub-databases are named as COG-500-1074, COG-250-1796, COG-100-2892. Here, COG-500-1074 indicates the COG database where each family has a minimum of 500 members and the number of families in this subset is 1074. The detailed description of these three subsets can be found in Table 1. For each subset, we have used a 3-fold cross-validation to train and test the models.

#### G protein-coupled receptor dataset

G protein-coupled receptor (GPCR) dataset is an important dataset for drug discovery as well as protein family classification. It provides a hierarchical classification with family, subfamily and sub-subfamily label for GPCR proteins. In this research, we used one of the biggest GPCR dataset, GDS [39], which consists of 8, 222 protein sequences divided into 5 families, 38 sub-families and 86 sub-subfamilies.

### Performance evaluation on COG dataset

In this part, we compare EnsembleFam with pHMM and DeepFam on the predictions of COG proteins.

**Fig. 1:**
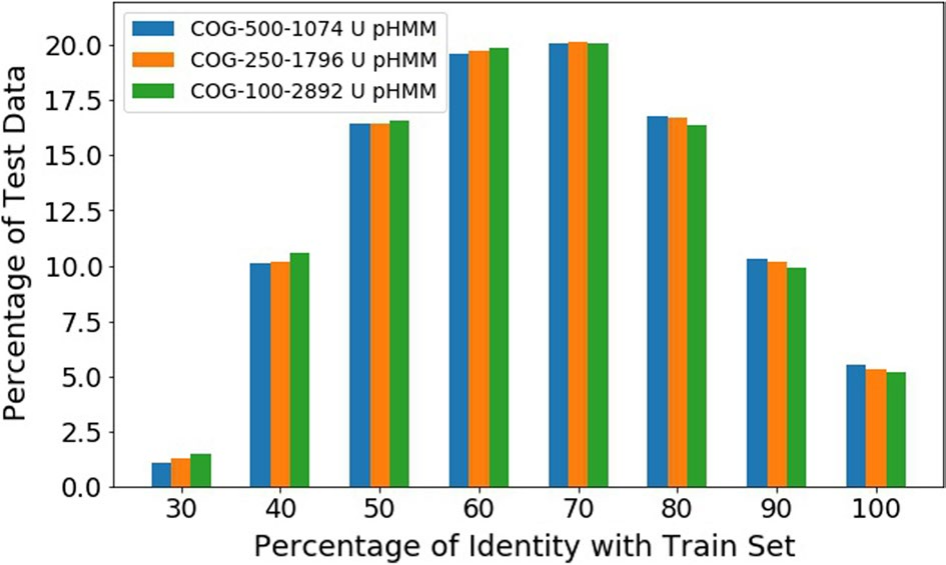
Homology between training and test set of COG dataset. The bars indicate the fraction of test data having identity less than or equal to the indicated value on the *x*-axis. For each fold of the dataset, the homology is calculated for test sequence against the training sequences and the seed sequences used to build pHMM feature models. For each identity percentage, the three different bars indicate the average of 3-fold of the three different subsets of COG dataset

#### Homology between training and test set

To run the experiment on our proposed approach, we use an independent test set, which is never used during any step of the training. For each dataset, we have divided the dataset into three equal splits and used two splits for training and one for the test. To show the independence of the test set, we try to find the homology between the training and test set. For each test sequence, we align them against the corresponding training sequences along with the pHMM seed sequences which we used to collect features. Then we collected the percentage of identity with any of those sequences using BLAST. We only collect a percentage of identity if the alignment length of the query sequence is greater than or equal to 100 residues, as a rule of thumb. In Fig. 1, we can see the average fraction of sequences in our test sets lying in different percentages of identity for COG dataset. For all the three datasets, more than 65% of test data fraction lie within an identity of less than or equal to 70% with the training set.

Although most of the test sequences have a considerable percentage of identity, we have a substantial amount of sequences with identity less than or equal to $40\%$. This portion of the test set, known as the twilight zone, is relatively harder to predict than others because they have a very low identity to the training set. Almost $12\%$ of the test set for all the three datasets are in this region.

#### Prediction accuracy for twilight zone proteins

One of the performance criteria we used to compare EnsembleFam with other methods, is prediction accuracy. In EnsembleFam, as we build one model for each family of each dataset (e.g. 1074 models for COG-500-1074 dataset), there can be multiple predicted labels for each test protein. To calculate the prediction accuracy we divided the test set in multiple subsets based on the number of predictions made by EnsembleFam.

**Table 2:** Performance comparison of different methods on the twilight zone sequences, i.e. sequences having less than $40\%$ identity is shown in this table

Dataset	Method	predCount = 1	predCount = 2	predCount = 3	predCount = 4	predCount = 5	predCount $> 5$
**Identity: **$0 < x \le 30$							
COG-500-1074	EnsembleFam	**72.07**	**81.00**	**82.82**	**84.96**	**85.33**	**85.27**
	pHMM	69.54	73.75	55.51	70.62	70.85	73.55
	DeepFam	57.14	54.52	49.90	46.92	43.64	35.94
COG-250-1796	EnsembleFam	72.84	**77.07**	**81.02**	**82.14**	**84.66**	**86.45**
	pHMM	**75.39**	73.82	73.84	71.02	67.44	72.43
	DeepFam	32.44	32.54	30.24	29.53	30.02	28.68
COG-100-2892	EnsembleFam	**75.24**	**79.55**	**81.21**	**80.63**	**82.05**	**88.95**
	pHMM	63.44	59.69	53.45	48.16	47.42	57.57
	DeepFam	27.30	26.13	25.54	27.62	24.83	25.36
**Identity: **$30 < x \le 40$							
COG-500-1074	EnsembleFam	**90.96**	**94.51**	**95.88**	**96.16**	**97.08**	**97.84**
	pHMM	62.22	61.20	88.95	87.38	85.19	85.85
	DeepFam	58.45	58.32	59.39	58.41	58.37	54.81
COG-250-1796	EnsembleFam	**91.54**	**95.19**	**95.52**	**95.95**	**96.62**	**97.73**
	pHMM	63.05	89.41	89.05	87.74	84.82	83.69
	DeepFam	47.09	48.38	50.12	51.09	50.73	48.78
COG-100-2892	EnsembleFam	**92.92**	**95.23**	**96.04**	**96.35**	**96.81**	**97.99**
	pHMM	87.07	87.78	86.08	84.04	80.16	81.69
	DeepFam	38.73	42.62	46.07	48.33	49.30	45.32

The best results are highlighted in bold font. The dataset is divided into six subgroups based on the number of predictions made by *EnsembleFam*. Using the column “predCount = 5” as an example, the accuracy in this table is computed as follows. For a protein, if EnsembleFam makes 5 function predictions for it, and one of these is correct, the protein is counted as correct in the column “predCount = 5”; if all 5 function predictions are incorrect, the protein is counted as a wrong prediction. For the same protein, regardless of how many function predictions are made by pHMM, as long as one of these is correct, the protein is counted as correct in the column “predCount = 5”; otherwise, the protein is counted as incorrect in the column. As for DeepFam, which makes exactly one prediction for each protein, the same protein is counted as correct in the column “predCount = 5” if and only if the sole DeepFam prediction for it is correct. All the accuracy value showed in the table is the average of 3-fold cross-validation

Here we concentrate on the prediction accuracy of EnsembleFam in the twilight zone of the test set. We divided the twilight zone sequences into two regions, one where the percentage of identity is $\le 30$ and the other with the percentage of identity $> 30$ and $\le 40$. The performance comparison of EnsembleFam with pHMM and DeepFam in these two regions is shown in Table 2. For each portion, the dataset is split into six subgroups based on prediction count. From Table 2, it is discernible that EnsembleFam prediction accuracy is almost 10–30% higher than other methods in almost all cases. EnsembleFam outperforms all other methods and its prediction accuracy is more than $90\%$ for all the subsets of COG dataset in the identity region 30–40%. Detail comparison of prediction accuracy for the whole COG dataset is provided in Additional file 1.

According to biological insights [3] predicting sequences in the twilight zone is much harder than other sequences. But EnsembleFam solves this problem with much higher accuracy than DeepFam and pHMM on the reported dataset.

#### ROC AUC score

To assess the performance of EnsembleFam, we calculated the Receiver Operating Characteristic (ROC) curve as another evaluation criterion. To get the ROC curve, the true-positive rate (TPR) and the false-positive rate (FPR) is calculated by varying the threshold of a learned model. TPR (also known as sensitivity) and FPR are computed using the following formula.

$$\begin{aligned} TPR= & {} \frac{TP}{P} = \frac{\text {True Positive}}{\text {Total number of Positive}} \\ FPR= & {} \frac{FP}{N} = \frac{\text {False Positive}}{\text {Total number of Negative}} \end{aligned}$$

**Fig. 2:**
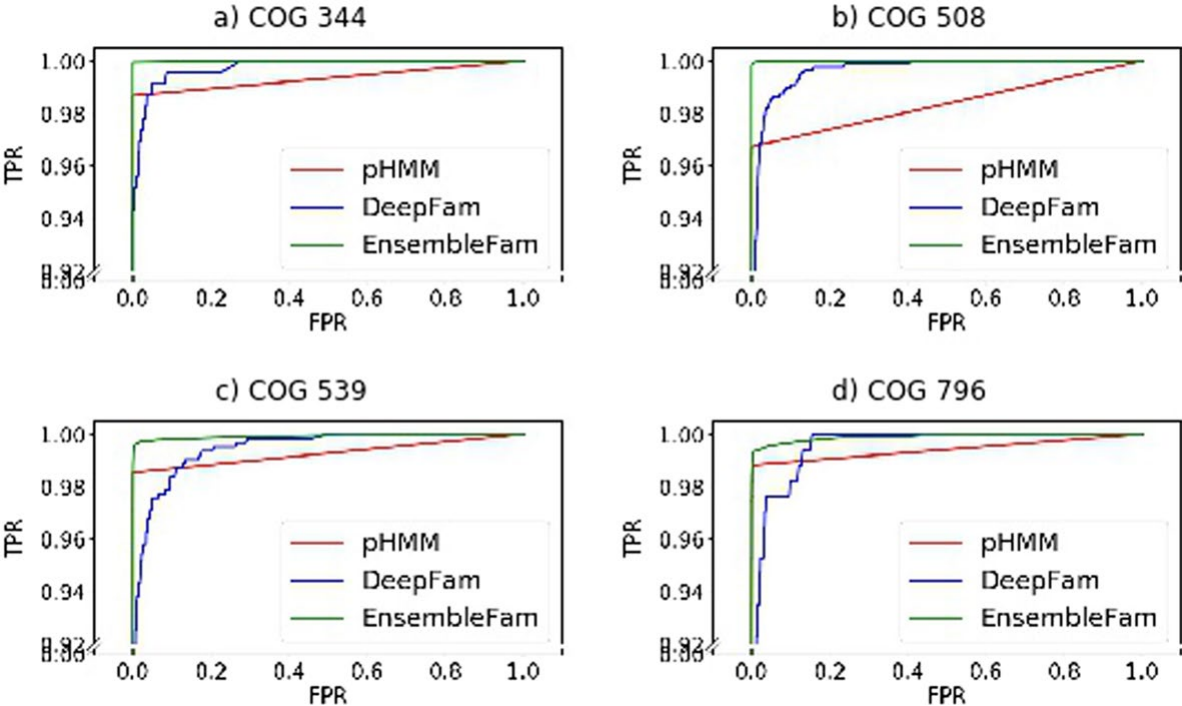
ROC curve for a few COG families from COG-500-1074 dataset. In each chart EnsmebleFam, DeepFam and pHMM are shown in different colors. It is clear that EnsembleFam performs better than other methods

In the ROC curve, the learned threshold is varied to observe how the model evolves if we want higher TPR or lower FPR. A good model will give us the highest TPR and lowest FPR for different thresholds, resulting in an inverted L-shape ($\Gamma$) ROC curve. To compare the performance of EnsembleFam with that of pHMM and DeepFam, we have plotted the ROC curve of a few families from the COG-500-1074 dataset in Fig. 2. From the figure, we can observe that the EnsembleFam curve is better $\Gamma$-shaped than others, i.e. the predicting power of EnsembleFam is more robust. As the performance of all three methods is pretty high, we plot the curve for TPR value 0.92 and higher to observe the clear difference. The ROC area under the curve (AUC) score is also calculated. The AUC scores for the four families of COG-500-1074 shown in Fig. 2 is listed in Table 3. From the AUC scores, EnsembleFam is better in all four families by a fair margin.

**Table 3:** ROC AUC score comparison of four families from COG-500-1074 dataset shown in Fig. 2

COG family	EnsembleFam	pHMM	DeepFam
COG 344	**0.999887**	0.993374	0.997536
COG 508	**0.999897**	0.983626	0.996149
COG 539	**0.999303**	0.992682	0.994334
COG 796	**0.999084**	0.994031	0.994851

The best results are highlighted in bold font

**Fig. 3:**
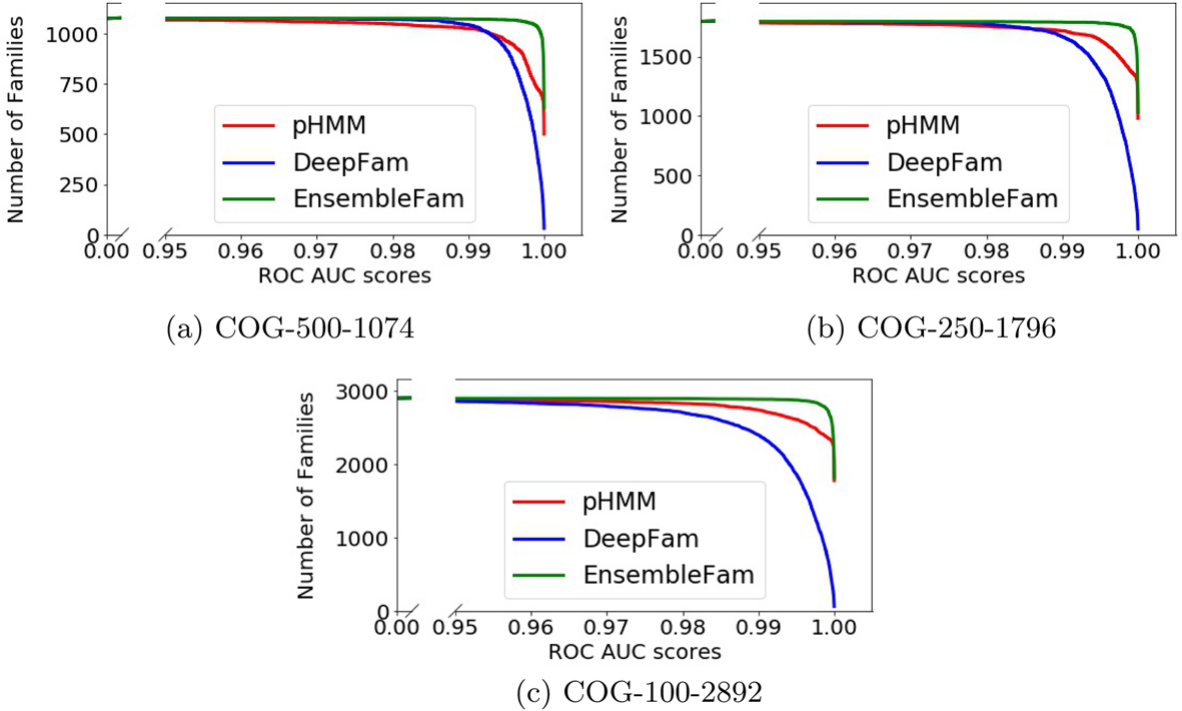
ROC AUC score comparison between EnsembleFam, DeepFam and pHMM on the three COG datasets. The $x-$axis shows the AUC score and the $y-$axis shows number of families in the respective dataset having AUC scores greater than or equal to the respective *x* value

Determining the AUC score for each family helps us choose the best model. For each dataset we have over a thousand families in our test set; e.g., we have 1074 families in COG-500-1074. To compare the AUC score of each family for the three datasets, we have plotted the AUC scores in Fig. 3. In this figure, for each dataset, *x*-axis indicates the AUC score and *y*-axis indicates the number of families having equal or higher AUC scores indicated in *x*-axis. The best model will have an AUC score of 1.0 for all the families, which will end up being a straight line at $y = \textit{number of families}$. All three methods have a very high AUC score for most of the families, the real difference is observed when we look at the higher AUC value ($> 0.95$). In Fig. 3, the number of families dropped for DeepFam and pHMM after reaching AUC score of 0.99. Whereas, EnsembleFam in all the three figures sustains longer and provides a higher AUC score for almost all the families which are closer to 1.0. This indicates that the performance of EnsembleFam is much better than DeepFam and pHMM in terms of the ROC AUC score on COG dataset.

#### ROC AUC score for new families

In this section, we evaluate the performance of EnsembleFam on test examples from new families that are not used in training. In our three dataset COG-500-1074, COG-250-1796, and COG-100-2892, the number of families is respectively 1074, 1796, 2892. As we divided the dataset based on the number of sequences above a certain threshold, the COG-100-2892 dataset includes all the families of COG-500-1074 and COG-250-1796. Therefore for this experiment, we used the test sequences from unique families of COG-100-2892 to test the models trained on COG-500-1074 and COG-250-1796. The COG-500-1074 models are trained on 1074 families, thus there are 1818 (2892 – 1074 = 1818) new families in COG-100-2892 which were never used while training models for COG-500-1074. Similarly, for COG-250-1796 models there are 1096 new families in COG-100-2892. We used the sequences of these families to test the performance of the models on new unknown family proteins. As none of the sequences belong to any of the trained families, the models should predict them as negatives.

**Fig. 4:**
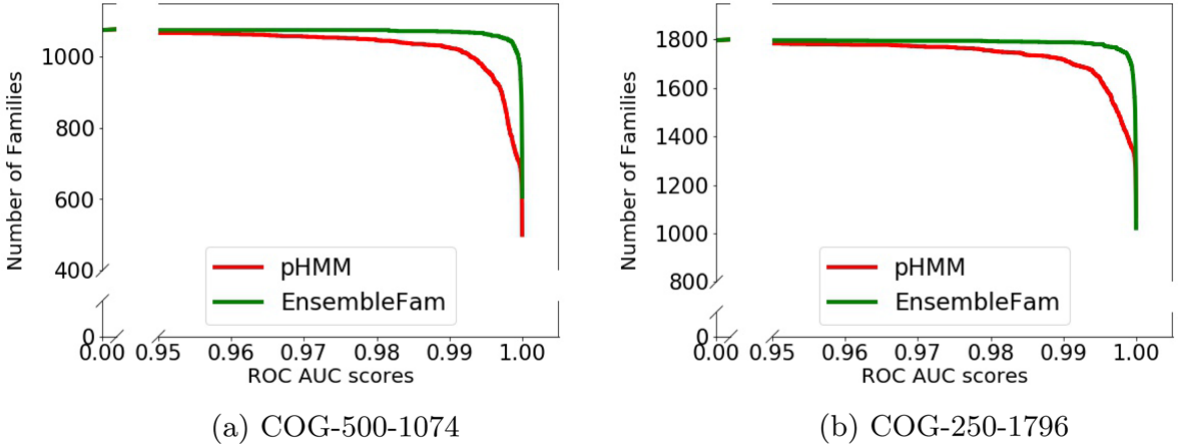
Test result of EnsembleFam and pHMM on new (unknown) family not used in training. For this, we used 1818 different families from COG-100-2892 to test the models of COG-500-1074, similarly 1096 different families for COG-250-1796. In the figure, $x-$axis indicates the ROC AUC score and the $y-$axis indicates number of families above that AUC score

We measured the ROC AUC score to assess the performance of EnsembleFam, similar to the previous section. We plotted the number of families versus AUC score curve in Fig. 4. In our test sequences, all the samples are negative, but we need positive samples as well to calculate the ROC AUC score. For this, we included the positive test sequences of the respective family along with these negative test sequences from new families. From Fig. 4 we can see EnsembleFam AUC scores are close to 1.0 for most of the families, whereas for pHMM the number of families drops after reaching an AUC score of 0.99. It shows that EnsembleFam is more robust to samples from new unknown families than pHMM. In this experiment, we have not included DeepFam as it cannot handle sequences outside of trained families. As DeepFam is a neural network-based multi-class classifier, it will always try to force the test sequence into one of the trained classes. But none of these sequences belong to any of the trained classes, i.e. DeepFam will end up predicting all of these novel sequences incorrectly. This is one of the drawbacks of DeepFam. Therefore, EnsembleFam is superior to both DeepFam and pHMM in this experiment as well.

### Performance evaluation on GPCR dataset

GPCR is a hierarchically classified dataset divided into family, sub-family, and sub-subfamily which is rather different from the COG dataset. For this dataset, we first build models for the leaf, i.e., sub-subfamilies; and then propagate the prediction to the roots, i.e., sub-family and family level, in a bottom-up approach. As the number of sequences in the GPCR dataset is much fewer compared to the COG dataset, we conducted a 10-fold cross-validation experiment for this dataset. All the results reported here are the average of 10-fold cross-validation.

#### Prediction accuracy for twilight zone proteins

Similar to the COG dataset, we kept separate one portion of the data for the test and calculated homology with the training set. Due to hierarchical classification, we experimented with the sub-subfamilies and later propagated it to the upper level. As the number of sequences is quite small, there are only a few sequences in the twilight zone for this dataset. We have around 800 test proteins in each validation set, and only 4–5% of them belong to the twilight zone, i.e., have $\le 40\%$ identity with the training sequence. This only gives us around 30–40 sequences in the twilight zone.

**Table 4:** Number of prediction made by pHMM and EnsembleFam for twilight zone proteins where $30 < \text {identity} \le 40$

Method	seq1	seq2	seq3	seq4	seq5	seq6	seq7	seq8	seq9	seq10	seq11	seq12	seq13	seq14
pHMM	57	54	58	17	13	7	7	56	50	50	38	1	1	54
EnsembleFam	1	0	0	2	0	0	3	1	1	1	1	2	2	1

For each test sequence, the maximum number of predictions can be 86, i.e, the sequence belongs to all sub-subfamilies. And the minimum number of predictions can be 0, i.e., the sequence does not belong to any of the sub-subfamily. DeepFam was not included in this comparison, as DeepFam always predict a single label irrespective of the number of families

**Table 5:** Prediction accuracy comparison of different methods on the twilight zone proteins

Method	Sub-subfamily	Sub-family	Family
**Identity**: $0 < x \le 30$			
pHMM	5.51	11.76	39.80
DeepFam	5.53	16.88	61.44
EnsembleFam	**30**.**92**	**45**.**15**	**65**.**45**
**Identity**: $30 < x \le 40$			
pHMM	14.74	21.72	**85**.**37**
DeepFam	22.38	37.18	73.40
EnsembleFam	**30**.**38**	**49**.**65**	65.46

Best results are highlighted in bold font. For pHMM and EnsembleFam, we removed the predictions where the number of prediction is $> 5$ and considered them as wrong prediction. For others, where the number of prediction is $\le 5$ and the true label is included within the predicted one, we consider as correct. For DeepFam, as it only predicts one label, if the predicted label is the same as true label then we consider it as correct. EnsembleFam outperforms other two method in almost all cases

For pHMM and EnsembleFam, we have one predictive model for each sub-subfamily whereas, DeepFam provides only one multi-class model for all 86 sub-subfamilies. As such, there can be more than one predicted label for each sequence for both pHMM and EnsembleFam. In Table 4, the number of predictions made by pHMM and EnsembleFam can be found for proteins with identity $> 30$ and $\le 40$. From Table 4, we can perceive that the number of predictions made by pHMM for a protein is around 50 (out of 86), which is approximately $60\%$ of the total number of families. Although the correct class label may be included in these predictions, these predictions might not be helpful for biologists. To make the prediction beneficial for biologists, we discarded the predictions where the prediction count is more than five, i.e., we consider those as wrong predictions while calculating the accuracy. The performance comparison of EnsembleFam with pHMM and DeepFam presented in Table 5, demonstrates that EnsembleFam is better than other methods in correctly identifying twilight zone proteins. Performance comparison for whole GPCR dataset can be found in Additional file 1.

## Conclusions

EnsembleFam, a protein family modeling technique using an ensemble approach and sequence homology information, is presented in this study. Different protein families can be modeled using this approach even if they have only a few (10 in this study) annotated proteins. Compared to state-of-the-art methods, EnsembleFam provides better prediction accuracy while resolving the disadvantages of those methods. EnsembleFam provides several beneficial characteristics. Firstly, EnsembleFam is more accurate than sequence homology-based and alignment-free methods. EnsembleFam provides one model for each family which correctly identifies the huge number of negative examples without having lots of false positives. Notably, EnsembleFam’s modeling technique is effective in correctly classifying proteins from the twilight zone. To tackle the problem of the growing number of unidentified protein sequences from various genome projects, EnsembleFam, a fast and more accurate modeling technique, will be very useful.

Alongside the benefits of EnsembleFam, there are several issues that are left as future work. As we know, there are a lot of proteins which do not have any domains or families assigned to them. The Pfam database provides a set of such proteins, known as domains of unknown function (DUFs) [40]. We wish to conduct extensive experiments on such proteins to find possible domains for DUFs, and then later validate them experimentally as part of our future work. As described earlier, EnsembleFam provides more than one prediction for a considerable fraction of test sets. But the true label is included among these predictions in almost all cases. Compared to other methods where only one prediction is made, EnsembleFam helps a biologist in identifying the correct label instead of the wrong one. Several approaches can be taken to identify a correct label from the multiple predictions like by aligning the query sequence with a few training sequences from the predicted classes. Two very popular and significant datasets, namely the COG dataset and GPCR dataset have been used in this study. Although EnsembleFam has shown comparatively better performance in identifying twilight zone proteins, the experiments were only conducted for single-domain protein sequences. There exists a vast number of unidentified multi-domain protein sequences. The state-of-the-art method pHMM can handle multi-domain proteins but the accuracy and precision can be improved. Thus, to address this problem and to model protein families with multiple domains a new study is necessary.

## Methods

In this study, we introduce EnsembleFam. EnsembleFam is a machine learning-based approach that uses three different Support Vector Machine (SVM) classifiers to infer the family of a protein from its sequence. In this approach, we build a single-class classifier for each family, i.e. a separate ensemble classifier for each family, which determines if an input sequence belongs to that corresponding family or not. EnsembleFam uses three different sets of features to train the three SVM classifiers. The features contain different similarity and dissimilarity measures among the families. From a raw protein sequence, respective features are collected using popular tools. These features are then passed to the three SVM classifiers to make predictions for each family and then a majority voting approach is taken to determine the final prediction for a sequence if it belongs to a certain family or not. We describe the architecture of EnsembleFam and how the models are trained in this section.

### EnsembleFam

#### Architecture

To build a model for a specific family, we first use Basic Local Alignment Search Tool (BLAST) [13] and profile Hidden Markov Model (pHMM) [14] to collect different features. These two tools use different techniques to calculate the sequence similarity of an input sequence to a given database of proteins of a family for which we want to build the model. We use these similarity scores of a family as a feature for our SVM classifiers. For each sequence, its similarity scores to all families are collected. That means each feature vector contains a score for each family indicating the similarity of the corresponding protein sequence with that family. For each protein family *x*, we divide the features into two categories: similarity and dissimilarity features.

- *Similarity feature* The sequence similarity score of a sequence to the family *x* is referred to as the similarity feature of the family *x*. A sequence that belongs to family *x* is expected to have a higher value for this similarity feature.
- *Dissimilarity feature* The sequence similarity of a sequence to families other than family *x* is referred to as the dissimilarity features of family *x*. A protein belonging to family *x* is expected to have lower values for the dissimilarity features of the family *x*.

This idea of using dissimilarity features along with the similarity features was implicit in SVM-pairwise [26]. Moreover, in SVM-pairwise, the features are collected for all possible pairs of the training sequences; i.e. for a given sequence, its similarity score to all members of its own family, and all members from the rest of families are calculated. In contrast, in EnsembleFam, for each sequence, only one similarity score is generated for each family, indicating its similarity to that family. Thus the size of an EnsembleFam feature vector is in the order of the number of protein families; this is much smaller than the size of an SVM-Pairwise feature vector which is in the order of the total number of reference proteins in all families.

We use both BLAST and pHMM to collect features from the sequences. The BLASTDB we use for BLAST is created from the training families (more details in Sect. Features) and provides both similarity and dissimilarity features for all families. For pHMM, we use predefined Hidden Markov Models (HMMs) from the Pfam database [15]. All these predefined HMM models mostly differ from our target protein families, but there might be a slight overlap between some protein families. As such, we name these features as pHMM features, which can be considered as a mixture of similarity and dissimilarity features. Once we have collected all the features, three base SVM classifiers are trained for each family using the following feature sets:

- *SVM model 1* Trained on pHMM features + similarity and dissimilarity features from BLAST
- *SVM model 2* Trained on pHMM features + only similarity features from BLAST
- *SVM model 3* Trained on only similarity features from BLAST

Note that, for each family, three such base classifiers are trained using the respective similarity and dissimilarity features of that family, which are used later to predict members of that family. As a result, for each protein sequence, we get three predictions indicating either the protein belongs to the respective family or not. A majority vote is then used to make the final ensemble prediction for that family.

#### Features

In EnsembleFam, BLAST and pHMM are used for generating similarity and dissimilarity features.

*BLAST features* Most of the features used to train our models are collected using BLAST. We use 10 reference sequence from each family (that we intend to build a model for) to create the BLAST database (BLASTDB). Let us assume we have *N* families in total, so our BLASTDB size will be 10*N*, i.e. each time a sequence is provided it will be compared against these 10*N* sequences. Each sequence is then run against the BLASTDB and the hits, scoring above a defined threshold, are reported in the BLAST output. From the BLAST output, we only consider one hit from one family, i.e. if a sequence hits multiple sequences of the same family we only consider the one with high scores and use it as the BLAST features for that family. By repeating this process, we collect BLAST features from all the families for each sequence. For each family (corresponding to a hit in BLAST output) we use three features to represent the similarity or dissimilarity of the given sequence to the respective family, which are:

1. **Bit-score:** The bit-score of an alignment is a normalized form of a raw alignment score. The raw alignment score is defined as the sum of substitution and gap scores along with the penalties [41]. Bit-scores are usually normalized using the scoring system; therefore scores from different searches can be compared with each other. The higher the bit-score of an alignment the better, i.e. the given sequence is more similar to the one in the database.
2. **E-value:** The e-value of an alignment is the expected number of hits, having an equal or better bit-score, that can be found by chance. Hence the lower the e-value the more significant the alignment is [41].
3. **Identity:** The percentage of aligned positions of two sequences having the same residue in the two sequences is known as identity [41]. Often in BLAST hits many of the sequences align with only a small portion of the sequence, as BLAST looks for local alignment. Some of these may have high scores and good e-values, yet they are not useful for our analysis as they have a low percentage of matches. In such a case, identity helps us differentiate between longer aligned sequences from the shorter one, which in turn helps us identify the correct member of the family.

**pHMM features:** While gathering the BLAST features we create our own database and collect both similarity and dissimilarity features for our model. But for profile Hidden Markov Models (pHMMs) we do not build our own HMM models to collect features, rather we use some predefined models from the Pfam database [15]. For each pHMM, we take two features (which are quite similar to the BLAST features):

1. **Bit-score:** The bit-score in pHMM is somewhat similar to the one in BLAST. Here, bit-score is defined as log-odds ratio score of the likelihood of the profile HMM with respect to the likelihood of the null hypothesis [42]. So bit-score can be written as,
  $$\begin{aligned} \text {bit-score, }S = \log _2 \frac{\text {likelihood of pHMM }}{\text {likelihood of null hypothesis}} \end{aligned}$$
2. **E-value:** The definition of the e-value is similar to the one we have seen earlier. An e-value is the number of hits expected to achieve a certain bit-score or higher by chance [42].

#### Training the models

Once we have collected all the features from the training sequences using BLAST and pHMM, the next step is to train the SVM classifiers for each family. We use a different subset of the features to train different classifiers. Here we discuss the feature vector size for each model:

- **SVM model 1 for family** *x* **:** The first model is trained on all the features we collected using both BLAST and pHMM. For a single sequence, we get 3 features (*bit-score, e-value, identity*) from each family using BLAST. If we have *N* family in total, then we get a total of 3*N* features from BLAST. Similarly, for each sequence we get 2 features (*bit-score, e-value*), so in total 2*M* features from pHMM where *M* is the number of pHMM models. In total, the feature vector size is $2M + 3N$ for the first model.
- **SVM model 2 for family** *x* **:** For the second model, we only use the similarity features from BLAST and pHMM features. For a single sequence, we only use the 3 features corresponding to the family *x* from BLAST and all the 2*M* features from pHMM. Hence, the feature vector size for this model is $2M +3$.
- **SVM model 3 for family** *x* **:** The third model is rather a simple and naive one compared to the other two. For this, we only use the similarity features from BLAST to train the model. So we have only 3 features corresponding to family *x* for each sequence in this model. Although this is quite a simple model, it does pretty well in predicting members of the family *x*.

We have all the features that we require to train the models for our target protein families. Each base SVM classifier for a family in EnsembleFam is a single-class classifier. But when we build a single-class classifier, other than the target protein family, all other protein families form the negative class and we need to incorporate the negative examples to train our classifiers. If the total number of families is *N* and each family has at least *d* reference proteins, that means we will have approximately $d\times (N-1)$ negative examples and only *d* positive examples. For example, if $N=1000$ and $d=200$ then the count for negative examples would be approximately 200,000 compared to 200 positive examples, which creates a huge imbalance in our training data. To avoid such a scenario in EnsembleFam, we only use 10 instances from each negative family as our training data which reduces the number from 200,000 to only 10,000, i.e. 20 times smaller in this example. Here we assume each family has only 200 examples, whereas in real data we have more and the number of families also increases a lot. So, to train our SVM models we follow this rule to reduce the number of negative examples compared to positive. As we can see from the above example, the ratio between positive and negative examples is still huge, and each classifier can gain very high accuracy (more than $90\%$) by just saying *NO* to all input. For our problem, it is more important to detect the positive examples, i.e., the member of a certain family than detecting the one who does not belong. For this reason, while training our SVM models we use a weighted classifier where a positive example is $9\text {x}$ the weight of a negative example. So the models try to classify the positive examples more correctly than the negative ones. Along with that, we have used linear kernel and squared hinge loss to train three base SVM classifiers (as mentioned above) for each family.

#### Ensemble prediction

We build one ensemble classifier for each family, which consists of three base SVM classifiers trained on a different subset of our feature set. Once we build all three base classifiers, the ensemble decision is taken by majority voting; i.e., if two or three of the SVM classifiers agree on a prediction then we provide that as our ensemble prediction for the input sequence. This can be defined as,

1 $$\begin{aligned} {\hat{y}} = mode\{\ C_1(\text {x}),\ C_2(\text {x}),\ C_3(\text {x})\} \end{aligned}$$

Here, $C_j$ is the *j*-th classifier and $\text {x}$ is the input sequence. The prediction ${\hat{y}}$ is made by majority (plurality) voting of each base classifier.

The above formula (Eq. 1) gives us a prediction for each input for each protein family, but we also need a score (or probability) for each prediction to calculate the area under the Receiver Operating Characteristic (ROC) curve. As the ensemble prediction is made by majority voting from the three base classifiers, the ensemble score is also calculated using the same idea. We use the following formula to calculate probability score for EnsembleFam,

2 $$\begin{aligned} Pr(ensemble) = (p_1\ p_2\ p_3) + (p_1\ p_2\ q_3) + (p_1\ q_2\ p_3) + (q_1\ p_2\ p_3) \end{aligned}$$

Here, $$p_i \in [0,1] : \text {prediction probability of classifier } i$$ and $$q_i \in [0,1] : q_i = (1-p_i)$$

In Eq. 2, we use the formula to incorporate majority voting in our ensemble probability calculation. The equation gives a high probability if and only if at least two of the classifiers provide high probability for the input sequence.

To test the performance of EnsembleFam, we used two different types of test set to better understand the outcome. The first type is for testing the performance of EnsembleFam on trained protein families. For this first type of test set, a 3-fold cross-validation is used; i.e., out of the three equally divided subsets of the dataset, two are used for training and the other subset is used for testing the model. The second type is for testing the performance of EnsembleFam on completely unseen (novel) protein families. For this type of test sets, protein families which are never used for training and their member proteins are used for testing. This second type of testing is necessary because, in a real deployment, EnsembleFam can be expected to encounter proteins from completely novel protein families.

### Existing protein family modeling methods used for performance comparison

We have used pHMM and DeepFam methods to compare the performance of EnsembleFam using different metrics like prediction accuracy, area under the ROC curve. In this section, we provide a brief explanation of these two methods.

#### Profile hidden Markov model (pHMM)

One of the most popularly used alignment-based method, profile Hidden Markov Model (pHMM), is used to compare the performance of EnsembleFam. To construct a pHMM model for a protein family, we first aligned the multiple sequences using Clustal Omega [43], and then these alignments are passed to HMMER [44] to build the model. In HMMER, hmmbuild is used to build the model and then hmmpress to index and compress it. Later, we used hmmscan to predict family for a given protein sequence based on e-value reported in the output. We used HMMER v3.2.1 with all the default parameters to construct the pHMMs for our evaluation.

#### DeepFam

DeepFam is a deep learning-based protein family modeling method recently introduced which reported a competitive performance with pHMM. We use this method to compare the predictive performance of EnsembleFam. DeepFam builds a multi-class classifier from the training data. DeepFam models are trained using graphics processing unit (GPU) from the training sequences with all the default parameters provided in the paper. For one dataset, one multi-class classifier is built using DeepFam, later these models are used to compare the performance.

## Supplementary Information

**Additional file 1: Table S1**. Prediction accuracy comparison of different methods on the whole COG test set. **Table S2, S3, S4**: Identity based performance for COG-500-1074, COG-250-1796 and COG-100-2892 dataset are shown respectively. **Table S5**: Prediction accuracy comparison of different methods on the whole GPCR test set. **Figure S1**: Homology between training and test set of GPCR. **Table S6**: Prediction accuracy comparison of different methods on the GPCR dataset based on identity.

## Data Availability

The datasets generated and/or analysed during the current study are available in the EnsembleFam repository, https://github.com/NeamulKabir/EnsembleFam.
