# Supplementary material for: EnsembleFam: towards more accurate protein family prediction in the twilight zone
Source: BMC Bioinformatics. 2022 Mar 14;23:90. doi: 10.1186/s12859-022-04626-w (PMC8919565; doi:10.1186/s12859-022-04626-w)
Supplement: Supplementary file 1 — Additional file 1: Table S1. Prediction accuracy comparison of different methods on the whole COG test set. Table S2, S3, S4: Identity based performance for COG-500-1074, COG-250-1796 and COG-100-2892 dataset are shown respectively. Table S5: Prediction accuracy comparison of different methods on the whole GPCR test set. Figure S1: Homology between training and test set of GPCR. Table S6: Prediction accuracy comparison of different methods on the GPCR dataset based on identity. [file 12859_2022_4626_MOESM1_ESM.pdf]

Supplementary material for EnsembleFam

More results for COG dataset

Prediction accuracy on the whole COG dataset: Table S1 shows the prediction accuracy comparison of different methods on the COG test set. We can observe from the table that EnsembleFam outperforms other methods in all cases.

Table S1: Performance comparison of *EnsembleFam* with other methods in terms of accuracy is shown in this table. For the three different datasets, each testset is divided into six subgroups based on the number of predictions made by EnsembleFam. For example, *predCount* $= n$ is the subset of test samples for which EnsembleFam predicts only n labels, and the accuracy is the fraction of instances (in this subset of samples) whose true labels are among the predicted labels. The accuracy for *pHMM* is also calculated similarly but usually pHMM predicts more labels for each subgroup than EnsembleFam. On the other hand, *DeepFam* predicts only one label for each label as it uses a multi-class deep neural network classifier. From the table, *EnsembleFam* dominates in all cases. All the accuracy values reported here are the average of 3-fold cross-validation and the test data fraction indicates the percentage of data range for different subgroup.

Method	predCount = 1	predCount = 2	predCount = 3	predCount = 4	predCount = 5	predCount > 5
COG-500-1074						
Test Data Fraction	8.95 – 20.91%	15.31 – 25.57%	17.15 – 19.87%	8.02 – 16.02%	12.35 – 12.88%	12.17 – 29.74%
pHMM	96.46	97.22	97.06	96.63	95.74	95.44
DeepFam	84.88	82.49	80.86	79.46	78.18	77.25
EnsembleFam	**98.14**	**98.59**	**98.73**	**98.87**	**98.87**	**99.13**
COG-250-1796						
Test Data Fraction	11.51 – 23.53%	17.52 – 20.54%	17.94 – 25.37%	7.05 – 15.22%	10.69 – 11.65%	13.01 – 26.28%
pHMM	96.44	97.08	96.89	96.13	94.88	95.04
DeepFam	72.29	71.60	71.22	71.28	71.20	70.48
EnsembleFam	**97.74**	**98.41**	**98.54**	**98.81**	**98.80**	**99.17**
COG-100-2892						
Test Data Fraction	19.65 – 30.06%	22.04 – 25.29%	16.25 – 18.83%	9.58 – 12.26%	5.78 – 7.9%	11.79 – 20.12%
pHMM	96.59	96.75	95.83	94.36	95.70	95.84
DeepFam	61.51	62.59	64.87	67.41	68.12	67.89
EnsembleFam	**98.01**	**98.44**	**98.71**	**98.82**	**98.96**	**99.37**

Homology based performance comparison for COG dataset: In this section, we provide the result for different COG subset based on different identity level. In the main manuscript, we have only shown performance for the twilight zone proteins ($0 <$ identity ≤ 40). In Table S2, S3, S4 identity based performance for COG-500-1074, COG-250-1796 and COG-100-2892 are shown respectively for all identity range.

Table S2: Identity based performance of different methods on COG-500-1074 testset. We divided the testset into five subset based on identity. In the table, the first column identity shows the percentage of the identity of the test set with respective train set.

COG-500-1074							
Identity	Method	predCount = 1	predCount = 2	predCount = 3	predCount = 4	predCount = 5	predCount > 5
$0 < x \leq 30$	EnsembleFam	**72.07**	**81.00**	**82.82**	**84.96**	**85.33**	**85.27**
	pHMM	69.54	73.75	55.51	70.62	70.85	73.55
	DeepFam	57.14	54.52	49.90	46.92	43.64	35.94
$30 < x \leq 40$	EnsmebleFam	**90.96**	**94.51**	**95.88**	**96.16**	**97.08**	**97.84**
	pHMM	62.22	61.20	88.95	87.38	85.19	85.85
	DeepFam	58.45	58.32	59.39	58.41	58.37	54.81
$40 < x \leq 50$	EnsmebleFam	**97.12**	**98.26**	**98.52**	**98.91**	**99.07**	**99.27**
	pHMM	90.77	93.22	93.45	92.65	90.90	91.53
	DeepFam	72.77	72.11	71.77	70.69	70.17	69.02
$50 < x \leq 60$	EnsmebleFam	**98.36**	**98.91**	**99.08**	**99.22**	**99.28**	**99.44**
	pHMM	93.24	95.13	94.95	94.33	92.37	93.33
	DeepFam	80.34	79.34	78.72	77.53	76.38	76.26
$60 < x \leq 100$	EnsmebleFam	**99.31**	**99.47**	**99.54**	**99.48**	**99.60**	**99.63**
	pHMM	97.50	97.65	97.34	96.71	95.78	95.68
	DeepFam	59.17	58.62	57.87	57.50	56.94	57.28

Table S3: Identity based performance of different methods on COG-250-1796 testset. We divided the testset into five subset based on identity. In the table, the first column identity shows the percentage of the identity of the test set with respective train set.

COG-250-1796							
Identity	Method	predCount = 1	predCount = 2	predCount = 3	predCount = 4	predCount = 5	predCount > 5
$0 < x \leq 30$	EnsembleFam	72.84	**77.07**	**81.02**	**82.14**	**84.66**	**86.45**
	pHMM	**75.39**	73.82	73.84	71.02	67.44	72.43
	DeepFam	32.44	32.54	30.24	29.53	30.02	28.68
$30 < x \leq 40$	EnsembleFam	**91.54**	**95.19**	**95.52**	**95.95**	**96.62**	**97.73**
	pHMM	63.05	89.41	89.05	87.74	84.82	83.69
	DeepFam	47.09	48.38	50.12	51.09	50.73	48.78
$40 < x \leq 50$	EnsembleFam	**96.98**	**98.19**	**98.52**	**98.89**	**98.99**	**99.31**
	pHMM	91.93	93.60	93.32	92.51	90.33	90.32
	DeepFam	62.07	63.38	63.97	64.61	65.28	63.91
$50 < x \leq 60$	EnsembleFam	**98.21**	**98.70**	**99.07**	**99.24**	**99.27**	**99.51**
	pHMM	94.01	95.12	94.79	93.91	92.18	92.74
	DeepFam	70.26	71.04	71.34	72.36	72.33	72.07
$60 < x \leq 100$	EnsembleFam	**99.09**	**99.38**	**99.48**	**99.54**	**99.54**	**99.69**
	pHMM	97.47	97.61	97.39	96.73	95.63	95.22
	DeepFam	54.90	54.56	54.46	54.96	55.43	55.46

Table S4: Identity based performance of different methods on COG-100-2892 testset. We divided the testset into five subset based on identity. In the table, the first column identity shows the percentage of the identity of the test set with respective train set. EnsembleFam

COG-100-2892							
Identity	Method	predCount = 1	predCount = 2	predCount = 3	predCount = 4	predCount = 5	predCount > 5
$0 < x \leq 30$	EnsembleFam	**75.24**	**79.55**	**81.21**	**80.63**	**82.05**	**88.95**
	pHMM	63.44	59.69	53.45	48.16	47.42	57.57
	DeepFam	27.30	26.13	25.54	27.62	24.83	25.36
$30 < x \leq 40$	EnsembleFam	**92.92**	**95.23**	**96.04**	**96.35**	**96.81**	**97.99**
	pHMM	87.07	87.78	86.08	84.04	80.16	81.69
	DeepFam	38.73	42.62	46.07	48.33	49.30	45.32
$40 < x \leq 50$	EnsembleFam	**97.36**	**98.44**	**98.83**	**98.97**	**99.06**	**99.49**
	pHMM	93.49	94.09	93.06	91.49	89.40	91.32
	DeepFam	52.82	55.73	59.21	61.82	62.55	60.84
$50 < x \leq 60$	EnsembleFam	**98.35**	**99.02**	**99.23**	**99.29**	**99.43**	**99.69**
	pHMM	95.11	95.49	94.40	92.96	91.33	93.58
	DeepFam	61.53	63.66	66.26	68.48	69.57	69.36
$60 < x \leq 100$	EnsembleFam	**99.11**	**99.38**	**99.50**	**99.57**	**99.68**	**99.80**
	pHMM	98.02	97.92	97.37	96.50	95.32	96.07
	DeepFam	49.61	50.06	51.78	53.74	54.28	54.21

More results for GPCR dataset

Prediction accuracy on the whole GPCR dataset: Table S5 shows the prediction accuracy comparison of different methods on the GPCR test set. For all three methods, the predictions made for sub-subfamilies were propagated to sub-family and family level to calculate accuracy. From Table S5, it is discernible that EnsembleFam outperforms other methods by quite a margin. For EnsembleFam, we can observe that the accuracy drops from sub-subfamily to family as we go higher hierarchically. As EnsembleFam is built as single-class classifier, there are more than one predictions made for each test sequence. When we propagate the predictions to upper level, those multiple predictions increase false positives. As a result, the accuracy drops a little as we calculate accuracy for higher levels. In spite of that, EnsembleFam performs better than other methods.

Method	Sub-subfamily	Sub-family	Family
EnsembleFam	**99.01**	**98.54**	**96.83**
pHMM	78.50	86.82	95.77
DeepFam	64.05	75.39	91.17

Table S5: Prediction accuracy comparison of different methods on the whole GPCR dataset.

Homology based performance comparison for GPCR dataset: In this section, we provide the result for different GPCR subset based on different identity level. Figure S1 shows the homology between training and test set of GPCR. In the main manuscript, we have only shown performance for the twilight zone proteins ($0 <$ identity ≤ 40). In Table S6 identity based performance is shown for all identity range.

Figure S1: Homology between training and test set of GPCR dataset. The bars indicate the fraction of test data having identity less than or equal to the indicated value on the x-axis. For each fold of the dataset, the homology is calculated for test sequence against the training sequences. Compared to COG dataset, most of the test data in GPCR have very high similarity with the training data.

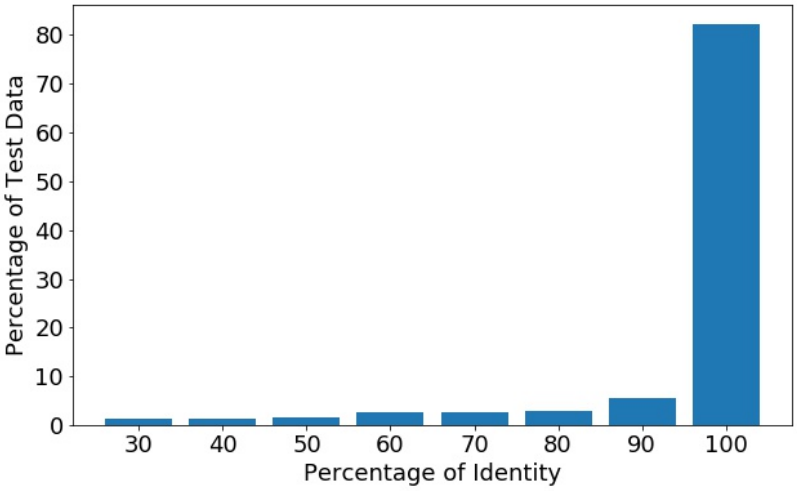

Table S6: Prediction accuracy comparison of different methods on the GPCR dataset based on identity. For pHMM and EnsembleFam, the predictions having predCount < 5 only those are counted as correct when the correct function is included in the predictions; predCount ≥ 5 are always counted as incorrect, even when the correct function is included in the predictions.. EnsembleFam outperforms other two method in almost all cases.

Method	Sub-subfamily	Sub-family	Family
**Identity**: $0 < x \leq 30$			
pHMM	5.51	11.76	39.80
DeepFam	5.53	16.88	61.44
EnsembleFam	**30.92**	**45.15**	**65.45**
**Identity**: $30 < x \leq 40$			
pHMM	14.74	21.72	**85.37**
DeepFam	22.38	37.18	73.40
EnsembleFam	**30.38**	**49.65**	65.46
**Identity**: $40 < x \leq 50$			
pHMM	14.87	18.68	**84.35**
DeepFam	26.05	38.13	68.11
EnsembleFam	**46.98**	**60.24**	70.91
**Identity**: $50 < x \leq 60$			
pHMM	19.25	30.08	81.49
DeepFam	23.66	38.35	64.17
EnsembleFam	**59.30**	**74.98**	**81.75**
**Identity**: $60 < x \leq 100$			
pHMM	18.02	28.18	99.07
DeepFam	67.24	76.02	92.29
EnsembleFam	**96.69**	**97.80**	**99.19**
